# Metabolomics approach revealed robust changes in amino acid and biogenic amine signatures in patients with schizophrenia in the early course of the disease

**DOI:** 10.1038/s41598-020-71014-w

**Published:** 2020-08-19

**Authors:** Madis Parksepp, Liisa Leppik, Kadri Koch, Kärt Uppin, Raul Kangro, Liina Haring, Eero Vasar, Mihkel Zilmer

**Affiliations:** 1 Department of Psychiatry, Institute of Clinical Medicine, University of Tartu, 31 Raja Street, 50417 Tartu, Estonia; 2 Psychiatry Clinic of Viljandi Hospital, 6 Pargi tee Street, 71024 Viljandi County, Estonia; 3 Psychiatry Clinic of Tartu University Hospital, 31 Raja Street, 50417 Tartu, Estonia; 4 Institute of Mathematics and Statistics, University of Tartu, 18 Narva mnt, 51009 Tartu, Estonia; 5 Institute of Biomedicine and Translational Medicine, Centre of Excellence for Genomics and Translational Medicine, University of Tartu, 19 Ravila Street, 50411 Tartu, Estonia

**Keywords:** Neuroscience, Biomarkers, Molecular medicine

## Abstract

The primary objective of this study was to evaluate how schizophrenia (SCH) spectrum disorders and applied antipsychotic (AP) treatment affect serum level of amino acids (AAs) and biogenic amines (BAs) in the early course of the disorder. We measured 21 different AAs and 10 BAs in a sample of antipsychotic (AP)-naïve first-episode psychosis (FEP) patients (n = 52) at baseline, after 0.6-year as well as after 5.1-year treatment compared to control subjects (CSs, n = 37). Serum levels of metabolites were determined with AbsoluteIDQ p180 kit using flow injection analysis tandem mass spectrometry and liquid chromatography technique. Elevated level of taurine and reduced level of proline and alpha-aminoadipic acid (alpha-AAA) were established as metabolites with significant change in AP-naïve FEP patients compared to CSs. The following 0.6-year treatment restored these alterations. However, further continuous 5.1-year AP treatment changed the metabolic profile substantially. Significantly elevated levels of asparagine, glutamine, methionine, ornithine and taurine, alongside with decreased levels of aspartate, glutamate and alpha-AAA were observed in the patient group compared to CSs. These biomolecule profile alterations provide further insights into the pathophysiology of SCH spectrum disorders and broaden our understanding of the impact of AP treatment in the early stages of the disease.

## Introduction

Schizophrenia (SCH) spectrum disorders are primarily diseases of the mind, affecting primarily mental, cognitive, emotional or behavioural functioning. What we tend to neglect is that psychotic disorder has always been a whole-body disease.

Clinical deterioration that occurs in SCH may begin in the prodromal phase and early identification and intervention may favourably alter the course and outcome of SCH^1^. To strengthen the effectiveness of interventions, a deeper scientific effort is needed to properly identify and characterize the early phase of the disease. The lack of standardised definitions of early SCH allows for considerable variation of patient groups between studies^2^. The term *first-episode psychosis* (FEP) is typically used to refer to an individual who has presented, been evaluated, and received treatment for the first abrupt or insidious onset of a psychotic episode associated with an SCH spectrum diagnosis^3^. According to the American Psychiatric Association’s definition, *early course* is the period after recovery from a first episode of SCH and extending up to the subsequent five years^4^. During these years clinical manifestations and biological characteristics of the disease can vary in type and severity over time. Besides, antipsychotic (AP) drugs given to treat the condition may relieve psychiatric symptoms but worsen the overall condition because of potential adverse effects.

In the last decade, high-throughput metabolomics technologies have provided additional comprehensive insights into the pathophysiological mechanisms of diseases. Metabolic profiles of serum may aid to understand disease progression and treatment effects^5^. In many recent studies, disturbances in the levels of several metabolites have been linked to the pathophysiology of SCH^6,7^. However, the molecular mechanisms of amino acids (AAs) and biogenic amines (BAs) serum levels associated with SCH along the continuum from FEP to the chronic stage of the disease remain elusive.

AAs are required as building blocks of proteins and peptides, some AAs are neurotransmitters (e.g. glutamate (Glu), glycine (Gly), serine (Ser)) or needed for the synthesis of several neurotransmitters and the maintenance of redox homeostasis in the organism^8^. BAs are mostly formed by decarboxylation of specific AAs. The synthesis of BAs is related to the uptake of their AA precursors and the levels of substrates in the brain are influenced by the blood concentration of these biomolecules. BAs serve several principal biological roles in the body (e.g. they are important messenger substances and regulators of cell function, they play an essential role in neurotransmission, neuromodulation, membrane stabilization, immunomodulation, and metabolism regulation). Altered concentrations of arginine (Arg), Glu, Gly, proline (Pro), Ser, isoleucine (Ile), valine (Val), and kynurenine (KYN) have been reported by many authors, but the results show inconsistencies in patients with SCH compared to control subjects (CSs)^7,9,10^. We have previously reported a significant alteration of AAs and their derivative BAs in serum samples of FEP patients^11^. According to the study, AP-naïve FEP patients had significantly higher levels of taurine and spermine, whereas the values of Pro, and alpha-aminoadipic acid (alpha-AAA) were diminished compared CSs after multiple testing correction. Also, the analysis revealed that 7-month AP treatment significantly affected the levels of Pro, histidine (His), taurine, KYN, alpha-AAA, acetylornithine (AcOrn), and spermine. However, the levels of these candidate biomarkers in FEP patients after treatment were in a similar range to those established for the control group^11^. A recent study by Cao et al. 2019 demonstrated that 8-week antipsychotic treatment caused an upregulation of cysteine (Cys), Pro, Arg, ornithine (Orn), and Glu in drug-naïve FEP patients and patients with SCH who had not taken APs for at least 4 weeks before inclusion into the study. Taken together, these studies suggest that changes in blood concentrations of AAs and their biogenic derivates (BAs) may affect susceptibility to psychotic experiences and provide new insights into the effect of AP treatment.

The present work outlined here aimed to broaden our previous results and to evaluate the effect of the early course of SCH spectrum disorders and applied AP treatments on free AAs and BAs profile in the serum of patients during a 5-year follow-up. This longitudinal study is the first to report the concentration of 21 AAs—alanine (Ala), Arg, asparagine (Asn), aspartate (Asp), citrulline (Cit), glutamine (Gln), Glu, Gly, His, Ile, leucine (Leu), lysine (Lys), methionine (Met), Orn, phenylalanine (Phe), Pro, Ser, threonine (Thr), tryptophan (Trp), tyrosine (Tyr), Val – and 10 BAs like AcOrn, alpha-AAA, asymmetric dimethylarginine (ADMA), creatinine, KYN, histamine, putrescine, symmetric dimethylarginine (SDMA), serotonin, and taurine in patients with FEP before and after AP treatment during 5 years. For evidence-based decision making in psychiatry practice, it is essential to know the long-term, natural course of SCH spectrum disorders and patients’ outcomes under care and treatment.

## Results

### General description of the study samples

Baseline demographic and clinical characteristics for study participants are shown in Table 1. There were no statistically significant differences between AP-naïve FEP patients and CSs in terms of age (*t*_(87)_ = 1.74, *p* = 0.09), gender (*χ*2_(1)_ = 2.33, *p* = 0.13), or mean values of BMI (*t*_(87)_ = -0.23, *p* = 0.82). In the patients’ group, AP treatment reduced psychopathology (BPRS) score significantly (*p* < 1e-06), but caused a significant increase in weight, BMI and waist circumference (*p* < 3e-05, *p* < 1e-06, and *p* < 1e-06, respectively).

**Table 1:** Characteristics of control subjects (CSs) and first-episode psychosis (FEP) patients at baseline (before treatment with antipsychotics (FEP_b_), after 0.6-year treatment (FEP_0.6-year_), and after 5.1-year treatment (FEP_5.1-year_) with antipsychotics.

*Characteristics*	Participants				Comparison between groups	
	CSs	FEP_b_	FEP_(0.6-year)_	FEP_(5.1-year)_	FEP_b_ and CSs	FEP_b,_ FEP_(0.6-year),_ and FEP_(5.1-year)_
Participants	37	52	44	37		
Age (years),(mean ± SD)	24.9 ± 5.3	27.0 ± 6.1	27.7 ± 6.5	32.0 ± 5.9	*t*_(87)_ = 1.74 ns	–
Women (%)	21 (57%)	21 (40%)	18 (41%)	14 (38%)	Χ^2^_(1)_ = 2.33 ns	–
Current cigarette smoker (n,%)	5 (14%)	17 (33%)	14 (32%)	18 (49%)	χ^2^_(1)_ = 4.27 *p* = 0.04	χ^2^_(2)_ = 1.33 ns
Weight (kg), (mean ± SD)	71.6 ± 15.0	70.2 ± 13.5	77.6 ± 16.2	85.8 ± 17.4^c^	*t*_(87)_ = -0.44 ns	*F*_(2)_ = 11.48 *p* < 3e−05
BMI (kg/m^2^), (mean ± SD)	23.0 ± 3.1	22.8 ± 3.0	25.4 ± 4.0^a^	27.8 ± 4.5^b,c^	*t*_(87)_ = -0.23 ns	*F*_(2)_ = 19.56 *p* < 1e-06
Waist circumference (cm), (mean ± SD)	80 ± 12	81 ± 10	87 ± 11	95 ± 11^b,c^	*t*_(89)_ = 0.53 ns	*F*_(2)_ = 17.46 *p* < 1e-06
BPRS score (mean ± SD)	–	49.9 ± 15.5	23.3 ± 12.7^a^	14.1 ± 10.6^b,c^	–	*F*_(2)_ = 87.31 *p* < 1e−06
AP dose (mean ± SD)	–	–	358 ± 162	418 ± 236	–	*t*_(36)_ = −1.28 ns

*BMI* body mass index, *BPRS* Brief Psychiatric Rating Scale, *AP dose* chlorpromazine equivalent dose of antipsychotics.

*ns* not significant (*p* ≥ 0.05).

^a^Statistically significant difference (*p* < 0.05) between patients before (FEP_b_) and after 0.6-year treatment (FEP_(0.6-year)_).

^b^Statistically significant difference (*p* < 0.05) between 0.6-year (FEP_(0.6-year)_) and 5.1-year treatment (FEP_(5.1-year)_).

^c^Statistically significant difference (*p* < 0.05) between patients before (FEP_b_) and after 5.1-year treatment (FEP_(5.1-year)_).

### AAS, BAs and their ratios alterations among patients with SCH spectrum disorder: at drug-naïve status, and after 0.6 years and 5.1 years of continuous AP treatment

Longitudinal associations between serum metabolites and the effects of AP drug treatment on disease status were tested by LME models based on 21 AAs and 10 BAs and their metabolically relevant ratio data. Patients data were compared to CSs after adjusting for covariates. For primary analyses, a set of two LME models was tested; both models use all the available data, but patient-specific determinants were only taken into account in the unrestricted models. Details of the models considered are given in Supplementary material Tables S1–S3. According to FDR adjusted *p*-value derived from ANOVA comparisons, unrestricted models provided a significantly better fit than the reduced model for the change in the levels of 12 AAs (Ala, Asn, Asp, Cit, Gln, Glu, His, Met, Orn, Pro, Trp and Val) and 5 BAs (alpha-AAA, ADMA, KYN, putrescine, taurine) over time, indicating significant differences between the behaviour of those metabolites between the patients and the control group. A similar outcome emerged when we compared candidate LME models of the metabolite ratios change (alpha-AAA/KYN, Asp/Asn, Glu/Gln, Orn/Arg, and Tyr/Phe) over time.

Thereafter, a series of LME regression models were conducted to test our main hypotheses.

### Comparison of AAs and BAs serum concentration between AP-naïve FEP patients and CSs

After correction for multiple testing, we identified 1 independent AA, 2 BAs, and 2 calculated metabolite ratios together with six covariates (age, gender, BMI, smoking status, differences between three different measurement time points) when differences in metabolite profiles were compared between AP-naïve FEP patients and CSs. Patients demonstrated a significant decline in the levels of Pro (*t*_*(71)*_ = -−3.87, *p* = 2e−04), alpha-AAA (*t*_*(71)*_ =  −4.56, *p* < 1e−04) as well as ratios of Tyr/Phe (*t*_*(71)*_ = −4.00, *p* = 2e−04) compared to CSs. On the contrary, FEP patients had a higher level of taurine (*t*_*(71)*_ = 7.65, *p* < 1e-04) and a higher value of Orn/Arg ratio (*t*_*(71)*_ = 3.69, *p* = 4e−04) at baseline than CSs (full details of the data generation and fitting procedures are given in Supplementary material Tables S4–S6).

### Comparison of AAs and BAs serum concentration between FEP patients after 0.6-year AP treatment and CSs

Examination of the levels of serum AAs, BAs and calculated metabolites’ ratios after 0.6-year treatment with AP drugs showed that all the aforementioned altered metabolite levels returned to levels comparable with CSs and none of the measured metabolite or calculated ratio showed any significant change in serum concentrations (a summary of the model parameter estimates is provided in Supplementary material Tables S4–S6).

### Comparison of AAs and BAs serum concentration between patients after 5.1-year AP treatment and CSs

After adjusting for potential confounders, the presence of the SCH spectrum disorder and continuation of APs were associated with significant alteration in 8 out of 31 measured AAs and their derivates levels as well as 4 out of 5 values of calculated metabolite ratios, as evaluated at 5.1 years. The AAs displayed a heterogeneous association pattern: disease and treatment continuation were related to a decrease in Asp (*t*_*(71)*_ = −5.26, *p* < 1e−04) and Glu (*t*_*(71)*_ =  −12.19, *p* < 1e−4), and an increase in Asn (*t*_*(71)*_ = 4.73, *p* < 1e−04), Gln (*t*_*(71)*_ = 9.22, *p* < 1e−04), Met (*t*_*(71)*_ = 9.74, *p* < 1e−04) and Orn (*t*_*(71)*_ = 3.47, *p* < 9e−04) levels. Among the BAs, there was a pronounced decline of alpha-AAA (*t*_*(71)*_ =  −6.25, *p* < 1e−04). In contrast, taurine (*t*_*(71)*_ = 5.35, *p* < 1e−04) concentration was increased in patients with a psychotic disorder after a 5.1-year period. Of the ratios, alpha-AAA/KYN (*t*_*(71)*_ =  −5.71, *p* < 1e−04), Asp/Asn (*t*_*(71)*_ =  −8.26, *p* < 1e−04), and Glu/Gln (*t*_*(71)*_ =  −12.24, *p* < 1e−04) values were decreased, but Orn/Arg (*t*_*(71)*_ = 5.43, *p* < 1e−04) value was increased in the patient group, relative to CSs, after 5.1 years.

It is worth to emphasize that 0.6-year AP treatment reversed significant alterations in the levels of taurine and alpha-AAA, and the Orn/Arg ratio, induced by first psychotic episode. However, during the 5.1-year continuous treatment, the beneficial effects of antipsychotic drugs on these metabolite levels disappeared and, after controlling for all study covariates, similar trends were observable as were evident at the pre-treatment status (see Supplementary material Tables S4–S6).

**Figure 1:**
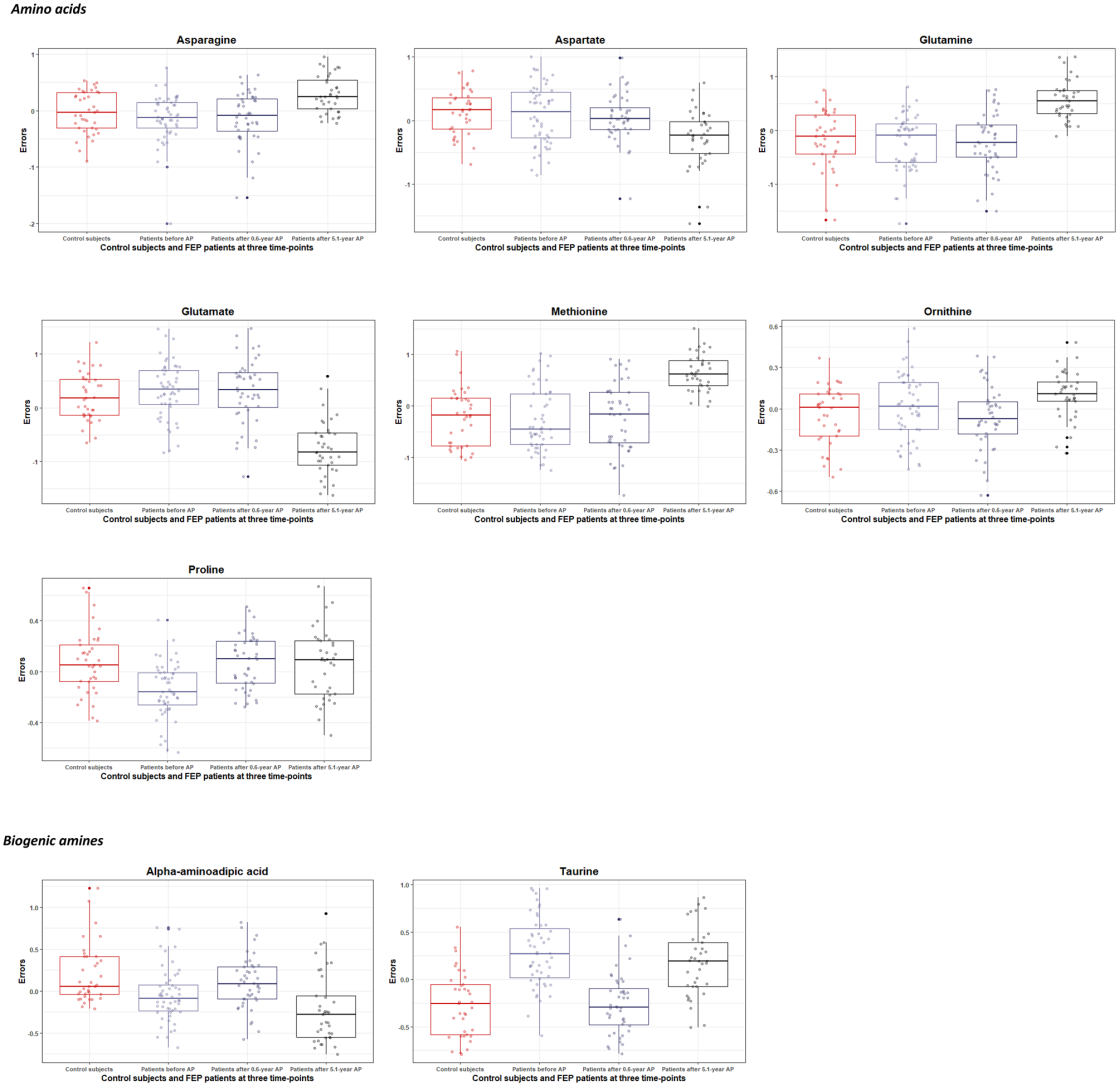
Boxplots of the statistically significant variation of the errors (residuals) of log-transformed amino acid and biogenic amine levels (derived by regressing out covariate effects) for control subjects and first episode (FEP) patients at baseline (before treatment with antipsychotics (AP)), after 0.6-year, and after 5.1-year treatment with AP. The solid horizontal line in each box represents the median. The area above and below the line represents the 50th to the 75th and the 25th to the 50th percentiles, respectively. The whiskers extend to the highest and lowest values contained within 1.5 times the interquartile range of the data. Each calculated error variance is represented as a dot.

**Figure 2:**
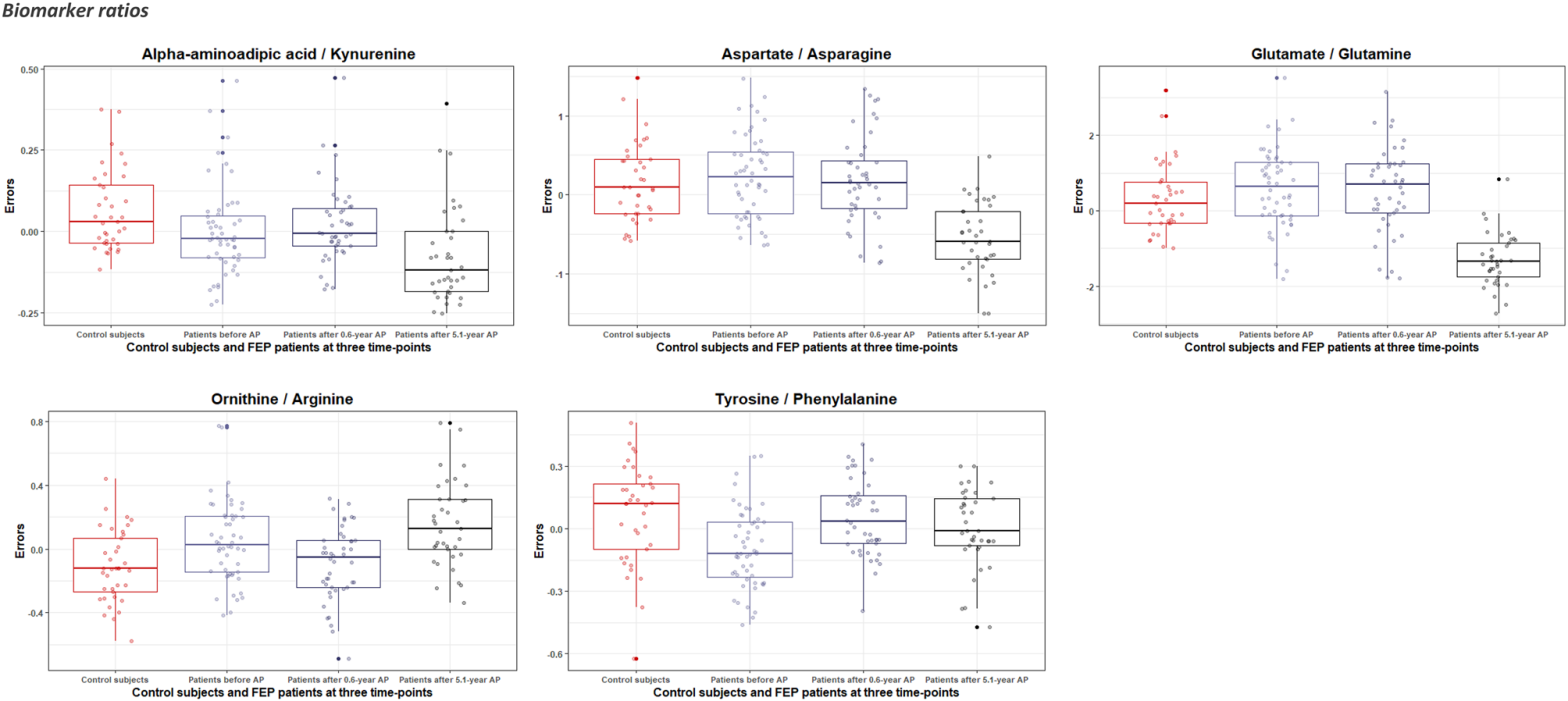
Boxplots of the statistically significant variation of the errors (residuals) of log-transformed biomarker ratios (derived by regressing out covariate effects) for control subjects and first episode (FEP) patients at baseline (before treatment with antipsychotics (AP)), after 0.6-year, and after 5.1-year treatment with AP.

Also, significant concentration differences (expressed in terms of residuals) in each measured circulating biomarker and their ratios between CSs and patients over three different time points were represented by box plots, as shown in Figs. 1, 2.

**Figure 3:**
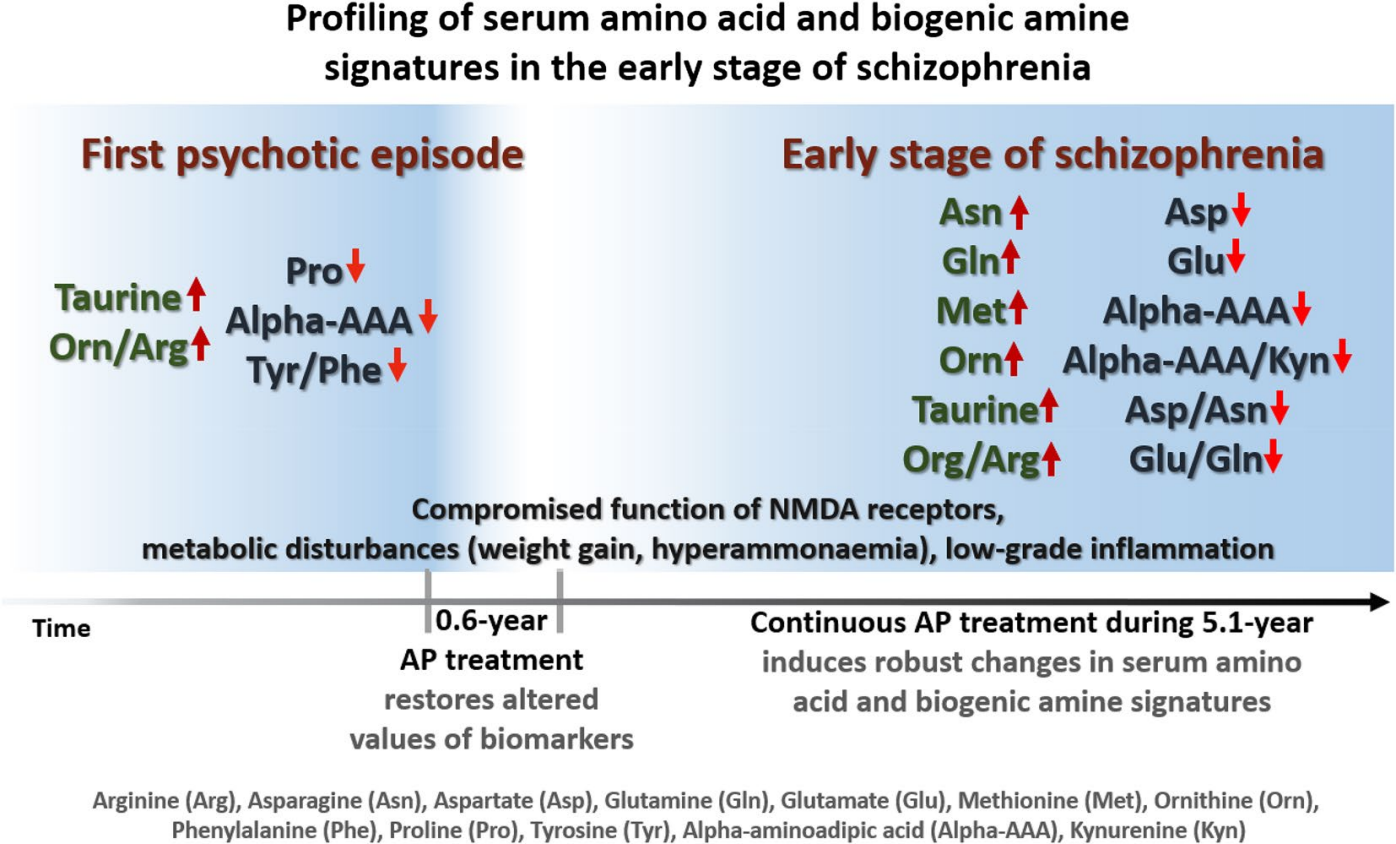
Schematic overview of the main results of the study.

A schematic summary of the main findings of this study is presented in Fig. 3.

## Discussion

Despite treatment advances over the past decades, SCH spectrum disorder may be a challenging condition to live with. Novel developments in biomarkers discovery are essential in modern health care. The use of metabolomics has recently emerged as a promising approach for the identification of potential diagnostic and treatment response candidate biomarkers for psychotic disorders. Endogenous metabolites, including AAs and BAs, are important in biological systems and are attractive candidates for understanding the disease entity^12^. Furthermore, the metabolomic profile of SCH has been shown to dynamically change with AP treatment^13^. Thus, longitudinal metabolomics profiling techniques are important to understand the disease or effect of the drugs across time and can provide an enhanced understanding of the underlying biology of the disease.

Comparison of AAs and BAs serum concentration between AP-naïve FEP patients and CSs revealed that among the most significant metabolites (together with six covariates: age, gender, BMI, smoking status, the time between measurements) that contributed to the discrimination between AP-naïve FEP patients and CSs, we confirmed an increase in taurine and a decline in alpha-AAA and Pro. Similarly to our previous studies^11,14^ taurine was the strongest nominator of the first frank psychotic episode in our present study population. Taurine has numerous cellular functions, including a central role as a neurotransmitter^15^, as a trophic factor in the central nervous system development^16^, as a brain osmoregulatory factor^17,18^, as a neuromodulator^19^ and as a neuroprotectant against the Glu induced excitotoxicity by reducing the Glu-induced increase of intracellular calcium level^20^. Studies have demonstrated that taurine has actions similar to those of the inhibitory amino acids Gly and gamma-aminobutyric acid (GABA), by producing a hyperpolarization of the neuronal membrane by modifying its permeability to chloride or potassium ions^21^. Furthermore, Chen et al. (2013)^22^ have demonstrated that taurine may directly interact with the Glu N-methyl-D-aspartate (NMDA) receptor diminishing the apparent affinity of the receptor to Gly in the presence of spermine. Increased levels of taurine, a molecule modulating the activity of NMDA receptors, probably reflects the compromised function of NMDA receptors in AP-naïve FEP patients^14^.

Besides that, reduced levels of alpha-AAA in our study may provide additional support to the idea of disturbed function of NMDA receptors. Alpha-AAA has been shown to inhibit the production of kynurenic acid (KYNA), a broad spectrum excitatory amino acid receptor antagonist, and thereby modulate KYNA function in the brain^23^. Also, it is a modulator of glucose homeostatic imbalance^24^.

In the present study, Pro level was reduced in AP-naïve FEP patients compared with CSs. However, some discrepancy exists between studies. Cai et al. (2012)^25^ did not find altered Pro level in AP-naïve patients with FEP compared to CSs, however, Garip and Kayir (2019)^26^ demonstrated that drug-naïve FEP patients had hyperprolinemia. Pro fulfils several of the classic criteria used to define neurotransmitters^27^ and previous results indicate that L-Pro is a weak agonist of the Gly receptor and at both NMDA and non-NMDA Glu receptors^28^. Additionally, Pro is an antagonist of glutamate decarboxylase, inhibiting the formation of GABA^29^. PRODH gene, responsible for the synthesis of proline dehydrogenase, degrading amino acid Pro, has been shown to belong to the risk genes of SCH^30^.

Tyr and Phe, if taken alone, did not discriminate FEP patients and CSs. However, the situation was different if the ratio between these molecules was calculated. Phe is biologically converted into Tyr, which is thereafter converted into dopamine and other catecholamines. Thus, alterations in the ratio between Tyr and Phe can be taken as a sign of compromised function of catecholaminergic neurotransmission. Previously, Wei et al. (1995)^31^ also confirmed that this ratio was significantly lower in AP-naïve FEP patients.

In the present study, Orn/Arg ratio discriminates FEP patients from CSs. The metabolic significance of the Orn/Arg ratio reflects arginase activity, an enzyme converting Arg into Orn and urea. Thus, the action of this enzyme is important both to produce Orn and to help to detoxify ammonia. It has been noted that hyperammonaemia may be a cause of psychosis in an adolescent^32^.

Comparison of AAs and BAs serum concentration between FEP patients after 0.6-year AP treatment and CSs demonstrated that the aforementioned aberrant metabolic signature was reverted to the level of CSs during the 0.6-year second-generation AP therapy. Concordantly, Cai et al. (2012)^25^ demonstrated that 6-week risperidone monotherapy restored plasma AAs profiles in FEP patients compared to CSs. However, this positive treatment effect, seen in our study, was combined with significantly increased BMI. It has been repeatedly reported that second-generation AP-treatment is associated with weight gain^33,34^. Unwanted effects of APs seem to be more pronounced at the onset of treatment (as soon as 8–12 weeks after initiation) in young AP-naïve patients with FEP^35^. Many factors, additionally to treatment, contribute to the elevation of BMI in patients with FEP, including effects of metabolic hormone signalling, genetic susceptibility, lifestyle change, and unhealthy food habits^36,37^. Furthermore, recent evidence points also to the importance of gut microbial composition in this process^38^.

Five-year AP treatment was associated with diminished psychopathology score measured by BPRS. Meanwhile, the body weight, waist circumference, and BMI of patients continued to increase over the follow-up period. At the metabolomics level, of the 31 individual metabolites tested, 8 (Asn, Asp, Gln, Glu, Met, Orn, alpha-AAA, taurine) demonstrated significant alterations when the data from patients were compared to CSs. Also, multiple pathways or biomolecule ratios demonstrated significant changes, with alpha-AAA/KYN, Asp/Asn, Glu/Gln, and Orn/Arg metabolism most significantly altered.

According to our results, the serum levels of Asn and Gln were increased and the levels of Asp and Glu were decreased as evaluated at the end of the 5-year follow-up. Moreover, in patients, the ratios between Asp/Asn, and Glu/Gln were significantly lower than in CSs. Asn and Asp have numerous biological functions in the human body, including participation in glyconeogenesis and brain functionality. Asp stimulates NMDA receptors compared to Glu only weakly^39^. Asn is involved in the metabolic control of cell functions in nervous tissue and it is synthesized from Asp and ammonia by asparagine synthetase^40^. Production of ammonia in brain tissue needs the presence of rapidly working elimination pathways in the brain. Conversion of pyruvate (Pyr) by alanine transaminase (ALT) into Ala is the main route to rapid binding of ammonia in the brain. As this reaction intensively consumes Glu, therefore, any excessive production of ammonia utilizes more Glu. This could be among the reasons why Glu was decreased in patients after the 5-year follow-up. Additionally, elimination of ammonia in the brain is controlled by the production of Gln and Asn, which may be one of the explanations for clearly elevated levels of Asn and Gln (and for declined levels of Asp and Glu, respectively) showed in our study after 5-year disease period and AP treatment. A decline of Asp levels can be partially explained by an intensification of its elevated targeting into glyconeogenesis as Asp belongs to the best substrates for this pathway due to direct conversion into key-metabolite of glyconeogenesis oxaloacetate. Besides that, Gln is one of the most abundant AAs, having an anaplerotic effect on the Krebs cycle and glyconeogenesis^41,42^.

Orn has some relation to the control of ammonia levels via the urea cycle. This cycle also consumes a certain amount of Asp to produce urea and Orn. In the present study, Orn level was significantly elevated, whereas the level of Arg remained unchanged in patients compared with CSs. At the same time, the Orn/Arg ratio was notably elevated both in AP-naïve patients and patients after 5-year follow-up period compared to CSs. Arginase is an enzyme that converts Arg into Orn and urea within the urea cycle. Therefore, Orn/Arg ratio shifts may reflect changes in the ammonia detoxification cycle. As toxic-free blood ammonia, can be transported to the brain through the blood–brain barrier^43^, it may cause pathophysiological changes in the central nervous system. A study by Popa et al. (2015)^44^ found that the average concentrations of ammonia in alveolar air appeared to distinguish patients with SCH from CSs.

Our results confirmed that after 5 years of AP treatment, the patients’ taurine level was increased again compared to CSs. In agreement with our results, Samuelsson et al. (2013)^45^ demonstrated a significantly elevated plasma level of taurine in patients with SCH. Furthermore, elevated levels of taurine in SCH were recently confirmed by Cao et al. (2019)^10^. Also, Shirayama et al. (2010)^46^ demonstrated that the level of taurine was significantly higher in the patients’ group than in CSs, as measured by proton magnetic resonance spectroscopy. Thus, elevated levels of taurine might offer a neuromodulatory defence mechanism against disturbed neurotransmitter homeostasis, particularly mechanisms involving the Glu NMDA receptor. Besides, an elevated level of taurine may, in part, be associated with a compensatory strategy against disrupted glucose and lipid metabolism, seen in patients with SCH and during the AP-treatment^47^. Beneficial effects of taurine appear to be mostly based on various protective effects against high glucose level^48^ and positive effects on lipid metabolism^49^.

Met level elevation, as it is taurine’s precursor through Cys, can be consistent with changes in taurine levels. There are psychiatric conditions like SCH, a bipolar disorder that, as well as AP treatment, can cause a shift in methylation/de-methylation balance in several biomolecules, e.g. in DNA^50^. These factors could explain elevated Met levels in association with five-year AP treatment.

Five-year disease course and AP-treatment were again associated with reduced levels of alpha-AAA. Alpha-AAA can modulate KYNA function^23^. KYNA is an antagonist at the Gly co-agonist site of the NMDA receptor^51^. It is produced along the KYN pathway during Trp degradation into KYN and is being considered a principal player in controlling glutamatergic and cholinergic synaptic transmission, and the coordination of immuno-modulation^52^. It is well-known that high levels of pro-inflammatory substances have been established in the blood and cerebrospinal fluid of SCH patients^53^. Inflammatory downstream cascades influence several pathways in the cells, including a shift in Trp metabolism toward the production of KYN instead of serotonin^54^. Previously, abnormally high KYN levels have been detected in the plasma of drug-naïve and medication-free patients with SCH^55^. To provide deeper insight into the KYN metabolic pathway, we calculated the ratio of alpha-AAA/KYN. According to this, the reduction of alpha-AAA concentration was accompanied by an increasing trend of KYN after 5-year AP treatment. Considering the possible interaction between alpha-AAA and KYN, it might be suggested that the imbalance in serum concentration of patients compared to CSs may indirectly contribute to the altered metabolism of Trp, which is accompanied by chronic inflammatory state.

Saleem et al. (2017)^7^ and Cao et al. (2019)^10^ have reported significant changes in the serum levels of Gly, Ile, Ser, His, Arg, and Cys in SCH patients compared to CSs. After using sophisticated and multi-level statistical approaches, our study failed to support the finding that the changes in Gly, Ile, Ser, His and Arg levels are statistically significant. Of course, contradictory findings between studies might be due to differences among samples and diagnostic subtypes. The lack of standardised definitions of early schizophrenia allows for considerable variation in patient groups between studies. Also, there is extensive variability of the phenotypes and biological features of the disorder concerning the pleiotropic nature of the underlying genetic and pathophysiological mechanisms of the disorder. Besides, different study designs, analytical techniques, and statistical methods are usually used, thus the findings need further validation in the future.

As we followed 36 patients for up to five years, we were able to assess dynamical changes in different types of molecules throughout the early course of psychotic disorder. Longitudinal design with data gathering at three-time points is a unique strength of this study. Important covariates (demographic factors, smoking status, follow-up time difference, and BMI) were taken into consideration as potential contributors and were controlled, giving the study another strength. Thirdly, the naturalistic approach allowed us to determine what kind of outcomes relating AA and BA levels patients are achieving in real-life settings. To our knowledge, it is the first study using targeted metabolomic profiling in given conditions.

A key limitation of the study is its relatively small sample size. Nevertheless, clinical metabolomics studies have led to successful discriminatory identification of metabolite profiles including approximately 30–50 subjects per group, based on case–control or time course studies^56^. Second, we collected data from CSs at one point in time and did not control their health condition or metabolite levels after the same follow-up period as was done for the FEP patients’ group. Third, since it was a naturalistic study, pharmacological treatment had no distinct restrictions. Therefore, usage of pharmacological agents was altered by clinically relevant circumstances but also may have been influenced by patient’s medication compliance as well, making it unattainable to determine the effect of specific AP drugs or chlorpromazine equivalents values.

## Conclusions

Many previous studies have linked disturbances of AA and BA levels to the pathophysiology of SCH. The current study profiled intra-individual changes in the levels of 21 AAs and 10 BAs using flow injection analysis tandem mass spectrometry as well as liquid chromatography technique in serial serum samples from FEP patients before and during 5.1-year treatment with APs compared to CSs. One AA and two BAs showed alterations in plasma levels, with taurine and alpha-AAA displaying the most significant changes when AP-naïve patients were compared to CSs. The following 0.6-year AP treatment restored the altered AA and BAs values and did not affect any other measured biomolecule concentrations when patients were compared to CSs. Further AP treatment up to an average of 5.1 years changed the metabolic profile substantially. Eight metabolites out of 31 individual biomolecules tested demonstrated significant change. Elevated levels of Asn, Gln, Met, Orn, taurine and diminished levels of Asp, Glu, and alpha-AAA discriminated patients from CSs. The longitudinal design, the broad range of targeted metabolic products, and consideration of important confounding factors in the present study provide advancement to the characterization of metabolism alteration in the early course of the psychotic disorder through a deeper understanding of AAs and BAs alterations in the patients with FEP before AP treatment, and after 0.6-year and 5.1-year AP treatment.

## Methods

### Participants

Patients with FEP (n = 52, 60% men) were recruited at the time of their first clinical contact for psychotic symptoms at the Psychiatric Clinic of Tartu University Hospital, Estonia. The inclusion criteria were as follows: patients with newly diagnosed, the duration of the untreated psychosis less than 3 years, no AP use before the study, male or female participants between 18 and 45 years old. Patients were allowed to receive benzodiazepines the night before the first blood collection at their psychiatrist’s discretion. The exclusions were as follows: patients had psychotic disorders due to another medical condition, and organic or drug-induced psychosis. FEP diagnoses were based on clinical interviews according to the International Classification of Diseases, Tenth Edition (ICD-10) (WHO, 1992) criteria^57^ and approved by two clinical psychiatrists. During the recruitment, patients’ diagnoses were F23.0 (*n* = 9), F23.1 (*n* = 11), F23.2 (*n* = 15), F23.3 (*n* = 2), F21 (*n* = 1), F20.09 (*n* = 11), and F20.39 (*n* = 3). All FEP patients were treated with AP medication. History of used APs was collected according to the reviews of patients’ medical charts. No restrictions were made in terms of usage of specific pharmacological substance due to a naturalistic and longitudinal study design. During the study, patients were treated with various doses and types of APs. Also, mood stabilizers, antidepressants or hypnotics were used according to clinically relevant circumstances.

Patients were examined prospectively. At an average of 0.6-year follow-up, the patients sample consisted of 44 patients (59% men), and at an average of 5.1-year follow-up, the sample comprised of 37 patients (62% men). During the monitoring period, the dropout rate was 29%. The main reasons for discontinuation were related to the patient’s decision to stop AP treatment or because they had changed their place of residence. The patient’s diagnoses at the second follow-up were: F20.0 (*n* = 27), F20.1 (*n* = 1), and F25 (*n* = 9).

The control group consisted of 37 subjects, who were recruited using advertisements. Of these mentally healthy participants, 43% were male. A more detailed description of the control group and principles of matching CSs to the patients are provided in our previous article published in 2018^11^. As it was a naturalistic study, substance abuse was not an exclusion criterion for either group. Nineteen patients (36%) had smoked cannabis before the FEP. Seventeen patients (45%) reported cannabis consumption during the monitoring period, and three of them (men) met the criteria for cannabinoid abuse disorder. One CS (3%) had tried cannabis at least once.

The study was approved by the Ethics Review Committee on Human Research of the University of Tartu, Estonia (initial approval No 177/T-2 and follow-up approval No 211/M-22) and carried out by The Code of Ethics of the World Medical Association. Written informed consent was provided by all participants. Partially, the same FEP and CSs groups have been characterized in our previous studies^11,14^.

### Procedure

Serum samples, and clinical and BMI data of the patients with a psychotic disorder were assessed at three-time points: on admission, at the first follow-up (mean duration 0.59 ± 0.06 years), and at the second follow-up (mean duration 5.15 ± 1.25 years). Fasting serum samples were collected from CSs and patients before and 0.6-year after the AP treatment was started between 1 June 2009 and 30 November 2014. The 5-year follow-up serum sample gathering began on 1 May 2013 and ended on 30 November 2017, the only patient group was comprised. Serum samples of participants were collected using standard venipuncture technique between 09:00 and 11:00 a.m. The severity of psychopathology was assessed using the Brief Psychiatric Rating Scale (BPRS)^58^. The BPRS consists of 18 symptoms and each item is measured on a seven-point Likert scale from “not present” to “extremely severe”. A total score was used as the outcome. Fasting blood samples and BMI data from CSs were collected cross-sectionally.

### Blood collection and clinical laboratory measurements

Blood (5 ml) was sampled in anticoagulant-free tubes and kept for 1 h at 4 °C (for platelet activation) before serum was isolated (centrifugation at 2000 rpm for 15 min at 4 °C). Collected serum samples were immediately frozen and stored at −20 °C for up to 2 weeks or at -80 °C for longer periods.

### Measurement of AAs and BAs

To assay serum level of AAs and BAs we applied the AbsoluteIDQ p180 kit (BIOCRATES Life Sciences AG, Innsbruck, Austria; https://www.biocrates.com/products/research-products/absoluteidq-p180-kit) using the flow injection analysis tandem mass spectrometry (QTRAP 4,500, Sciex, Framingham, MA, USA) as well as liquid chromatography (Agilent 1,260, Waldbronn, Germany) tandem mass spectrometry technique. The assays were performed according to the manufacturer’s manual UM-P180. Next, all metabolites were measured via a 2-stage process (in 2014, and 2017), by the same specialist of mass spectrometry laboratory, using the same methodology, equipment and equipment calibration techniques and laboratory tests were performed following standard procedures and quality controls. For quality control, we used a widely applied and well-validated commercial protocol. The MetIDQ software provided by Biocrates was used for quality assessment, data evaluation, and quantification of the metabolites of the FIA-measurements. Quality control samples, as well as samples for calibration curves, are part of the kit. The curves are built and performance of quality controls checked for each 96-well plate batch. The RSD is in the range from 0.5–13% depending on the metabolite. Since it is a commercial kit, which has been validated before, we see no reason to list all the technical details. However, we also analyzed our data on the basis according to special study on plasma sample pre-analysis handling 4 key factors (hemolysis, the temperature immediately following blood collection, time from collection to cooling, long-term storage temperature) must be met during pre-handling serum sample to measure concentrations of amino acids^59^. All these rules have been followed by us. Also, using detailed analysis focused on the long-term stability of samples^60^ we carefully checked all measured metabolites values regarding also such aspects. Data quality was checked based both on the level of detection and the level of quantification (LOD or LLOQ). All measured AA values were higher than LLOQ. ADMA, creatinine, KYN, serotonin, and taurine values were higher than LLOQ, and Ac-Orn, alpha-AAA, histamine, SDMA, putrescine values were at least 1.5 to 3 times higher than LOD. Average values of all measured AAs and BAs are given in the Supplemental material Table S1 and Table S2.

### Statistical analyses

All data were checked for normality of distribution using the Shapiro–Wilk test. Normally distributed data (age, weight, BMI, waist circumference) were analyzed using the Student’s *t*-test or repeated measure ANOVA, and mean differences were tested with Scheffé post hoc test. Dichotomous data (gender and smoking status) were analyzed using the chi-square test. To examine the alterations of putative biomarker levels between CSs and FEP patients over time, linear mixed-effects models (LME) were used. This modelling approach is flexible enough to account for natural heterogeneity in the population, to cope with unbalanced data sets, and to manage effectively drop-out and missing data^61^. Repeated measurements were handled by including a random intercept for participants in the model (since patients have different baseline values) and by allowing time-dependent correlations between different measurements of each patient. The model intercept was set to represent putative biomarker levels of the control group and variables’ coefficients were compared to this intercept. Each set of analysis was adjusted for potential confounders: gender, age at the first visit, BMI and smoking status, and the time difference between the visits (time difference between expected time and given time). However, these covariables were not of primary interest in this study. Time dependence between measurements of each patient was modelled by continuous autoregressive correlation structures of order 1 and models were fitted by maximum likelihood method. The candidate biomarker data was log-transformed before analysis to reduce the heterogeneity of variance commonly seen with metabolic data. Also, several biomarker ratios were calculated as indicators of metabolic processes.

First, to identify dependent variables which behave differently in the case of patients and the control group, we fitted two nested models to the data (i.e. reduced model with dependent variables with no patients’ specific independent variables compared to a more complex model with added terms allowing for the possibility for the value of the dependent variable to depend on patients type of visit and time between visits). The models were compared by the likelihood ratio test, where the false discovery rate (FDR) procedure was used for multiple testing corrections. Thereafter, the estimates from the LME analyses (fixed effects) were used to establish patients’ biomarker profile alterations at three different time points. As we ran several LME models in parallel, we adjusted the *p*-values by controlling the false discovery rate (FDR) at 25% using the Benjamini–Hochberg procedure^62^. We considered *p*-values (two-tailed) < 0.001 to be statistically significant while selecting the metabolites with the most pronounced change. The full models described above include only main effects.

The R statistical language version 3.5.0^63^ package *nlme*^64^ and ANOVA-type diagnostic test were used to perform analysis of the relationship between candidate biomarker levels differences among CSs and patients at three-time points, and Statistica software (StatSoft Inc., 13th Edition)^65^ for Windows was used for other analyses. The visualization of error variances obtained from regression results was done using R software package *ggplot2*^66^. Residuals or errors terms were assumed to be independent among individuals but dependent within each participant.

## Supplementary information

Supplementary information.

## Data Availability

The raw metabolomic data generated and analysed during the current study are available in the Figshare repository, https://doi.org/10.6084/m9.figshare.12640370. The full dataset that supports the findings of this study is available from the corresponding author (LH) on request.
