# Supplementary material for: Metabolomics approach revealed robust changes in amino acid and biogenic amine signatures in patients with schizophrenia in the early course of the disease
Source: Sci Rep. 2020 Aug 19;10:13983. doi: 10.1038/s41598-020-71014-w (PMC7438522; doi:10.1038/s41598-020-71014-w)
Supplement: Supplementary file 1 — Supplementary information. [file 41598_2020_71014_MOESM1_ESM.pdf]

*Supplementary material*

**Metabolomics approach revealed robust changes in amino acid and biogenic amine signatures in patients with schizophrenia in the early course of the disease**

Madis Parksepp, Liisa Leppik, Kadri Koch, Kärt Uppin, Raul Kangro, Liina Haring*, Eero Vasar, Mihkel Zilmer (Corresponding author* Liina.Haring@kliinikum.ee)

**Table S1.** Serum mean levels, standard deviations (SD), and range of amino acids (micromoles) for control subjects (CSs, n=37), first-episode psychosis (FEP) patients at baseline (before treatment with antipsychotics, $FEP_b$, n=52), after 0.6-year treatment ($FEP_{0.6\text{-year}}$, n=44), and after 5.1-year treatment ($FEP_{5.1\text{-year}}$, n=37) with antipsychotics. Comparison between reduced and unrestricted models, *p*-values have been multiple test corrected according to false discovery rate (FDR) method. The unrestricted regression model is designated as True (i.e. more complex set of predictor variables explained more effectively biomarker level alterations over time) or False (i.e. unrestricted model did not provide more explanatory power than a simple one).

| Regression equations for estimating biomarker level or biomarkers ratio alterations. | |
|---|---|
| Reduced model | log(biomarker)~Age+BMI+Gender+Smoking+(1\|Patient) |
| Unrestricted model | log(biomarker)~Age+BMI+Gender+Smoking+Visit+Time1+Time2+(1\|Patient) |

BMI = body mass index, Visit = biomarker measurements at three time points in patients' group, Time1 = time difference between $FEP_b$ and $FEP_{0.6\text{-year}}$, Time2 = time difference between $FEP_{0.6\text{-year}}$ and $FEP_{5.1\text{-year}}$, (1|Patient) = random effects of patients.

| *Amino acids* | CSs Mean ± SD (range) | $FEP_b$ Mean ± SD (range) | $FEP_{(0.6\text{-year})}$ Mean ± SD (range) | $FEP_{(5.1\text{-year})}$ Mean ± SD (range) | Comparison between models | |
|---|---|---|---|---|---|---|
| | | | | | Adjusted *p*-value | True (T) or false (F) |
| Alanine | 425 ± 118 (232 – 716) | 387 ± 112 (206 – 673) | 462 ± 134 (290 – 750) | 520 ± 171 (289 – 953) | 0.03 | T |
| Arginine | 163 ± 36.1 (94.1 – 225) | 144 ± 32.6 (75.9 – 216) | 155 ± 35.5 (93.0 – 263) | 143 ± 49.8 (72.5 – 327) | 0.08 | F |
| Asparagine | 37.2 ± 13.0 (15.0 – 65.2) | 33.8 ± 12.6 (4.80 – 83.3) | 35.7 ± 14.1 (8.06 – 75.5) | 53.2 ± 17.4 (30.2 – 87.4) | 2e-07 | T |
| Aspartate | 35.3 ± 10.7 (15.9 – 65.2) | 33.8 ± 14.3 (10.6 – 62.9) | 29.9 ± 11.5 (7.86 – 57.4) | 19.0 ± 7.14 (5.11 – 39.6) | 7e-06 | T |
| Citrulline | 27.8 ± 8.37 (11.0 – 48.9) | 22.5 ± 5.77 (12.1 – 38.1) | 25.0 ± 6.66 (11.5 – 39.4) | 29.1 ± 13.7 (14.1 – 76.7) | 9e-03 | T |
| Glutamine | 342 ± 153 (76.5 – 683) | 328 ± 148 (80.4 – 813) | 365 ± 176 (103 – 810) | 854 ± 308 (495 – 1760) | 4e-18 | T |
| Glutamate | 216 ± 99.2 (114 – 550) | 250 ± 116 (59.6 – 627) | 241 ± 121 (57.2 – 529) | 74.1 ± 40.1 (18.7 – 172) | 1e-25 | T |
| Glycine | 257 ± 72.1 (123 – 443) | 278 ± 65.1 (153 – 474) | 287 ± 81.9 (149 – 597) | 297 ± 81.5 (187 – 518) | 0.41 | F |
| Histidine | 94.0 ± 20.9 (58.3 – 138) | 86.4 ± 14.8 (61.5 – 135) | 97.7 ± 16.1 (73.3 – 136) | 107 ± 21.1 (66.4 – 157) | 9e-03 | T |
| Isoleucine | 92.2 ± 30.2 (50.1 – 179) | 86.8 ± 22.7 (42.7 – 158) | 95.4 ± 29.5 (43.9 – 190) | 96.1 ± 28.0 (46.4 – 164) | 0.95 | F |
| Leucine | 176 ± 68.9 (79.6 – 409) | 166 ± 47.6 (73.0 – 279) | 174 ± 56.8 (85.5 – 364) | 200 ± 78.4 (77.0 – 443) | 0.69 | F |
| Lysine | 203 ± 46.4 (107 – 309) | 205 ± 51.8 (117 – 314) | 217 ± 56.0 (103 – 399) | 231 ± 55.6 (124 – 393) | 0.69 | F |
| Methionine | 10.2 ± 6.37 (4.43 – 35.2) | 9.89 ± 6.36 (3.95 – 26.3) | 12.0 ± 7.38 (3.23 – 33.5) | 27.7 ± 7.76 (13.2 – 43.0) | 1e-18 | T |
| Ornithine | 55.8 ± 17.2 (23.4 – 91.4) | 66.7 ± 25.2 (30.7 – 143) | 63.3 ± 20.0 (28.4 – 125) | 81.5 ± 22.1 (39.9 – 136) | 8e-03 | T |
| Phenylalanine | 71.0 ± 19.7 (38.2 – 115) | 70.7 ± 12.5 (41.8 – 101) | 68.9 ± 15.2 (38.2 – 108) | 77.0 ± 20.1 (43.6 – 134) | 0.44 | F |
| Proline | 229 ± 79.2 (123 – 479) | 184 ± 55.5 (83.3 – 381) | 237 ± 55.4 (140 – 362) | 261 ± 91.2 (111 – 459) | 7e-05 | T |
| Serine | 170 ± 52.3 (69.3 – 363) | 174 ± 43.9 (99.4 – 293) | 168 ± 34.4 (115 – 253) | 159 ± 55.2 (88.9 – 346) | 0.69 | F |
| Threonine | 165 ± 60.2 (74.1 – 373) | 144 ± 35.0 (71.6 – 214) | 160 ± 38.1 (71.0 – 280) | 151 ± 62.3 (82.7 – 430) | 0.44 | F |
| Tryptophan | 74.2 ± 17.0 (32.8 – 120) | 66.9 ± 16.7 (30.3 – 120) | 71.7 ± 18.4 (34.2 – 121) | 82.4 ± 25.9 (46.1 – 157) | 0.02 | T |
| Tyrosine | 73.0 ± 27.9 (33.7 – 159) | 60.7 ± 13.8 (35.8 – 99.8) | 70.7 ± 19.4 (40.6 – 121) | 77.2 ± 23.6 (40.4 – 138) | 0.09 | F |
| Valine | 233 ± 62.1 (126 – 401) | 219 ± 61.7 (112 – 413) | 244 ± 67.0 (136 – 448) | 305 ± 83.7 (155 – 490) | 7e-03 | T |

**Table S2.** Serum mean levels, standard deviations (SD), and range of biogenic amines (micromoles) for control subjects (CSs, n=37), first-episode psychosis (FEP) patients at baseline (before treatment with antipsychotics, $FEP_b$, n=52), after 0.6-year treatment ($FEP_{0.6\text{-year}}$, n=44), and after 5.1-year treatment ($FEP_{5.1\text{-year}}$, n=37) with antipsychotics. Comparison between reduced and unrestricted models, *p*-values have been multiple test corrected according to false discovery rate (FDR) method. The unrestricted regression model is designated as True (i.e. more complex set of predictor variables explained more effectively biomarker level alterations over time) or False (i.e. unrestricted model did not provide more explanatory power than a simple one).

| ***Biogenic amines*** | **CSs** Mean ± SD (range) | **$FEP_b$** Mean ± SD (range) | **$FEP_{(0.6\text{-year})}$** Mean ± SD (range) | **$FEP_{(5.1\text{-year})}$** Mean ± SD (range) | **Comparison between models** | |
|---|---|---|---|---|---|---|
| | | | | | **Adjusted p-value** | **True (T) or false (F)** |
| Acetylornithine | 0.68 ± 0.36 (0.18 – 2.03) | 0.69 ± 1.24 (0.00 – 8.98) | 0.67 ± 0.47 (0.00 – 2.63) | 0.74 ± 0.68 (0.00 – 2.68) | 0.95 | F |
| Alpha-aminoadipic acid | 0.90 ± 0.40 (0.45 – 1.98) | 0.55 ± 0.34 (0.00 – 1.34) | 0.75 ± 0.38 (0.00 – 1.54) | 0.40 ± 0.45 (0.00 – 1.59) | 1e-06 | T |
| Asymmetric dimethylarginine | 0.42 ± 0.08 (0.19 – 0.60) | 0.44 ± 0.10 (0.28 – 0.67) | 0.44 ± 0.09 (0.29 – 0.63) | 0.58 ± 0.19 (0.26 – 1.19) | 0.008 | T |
| Creatinine | 69.4 ± 18.4 (35.0 – 112) | 68.3 ± 16.2 (39.1 – 123) | 69.7 ± 17.6 (42.0 – 124) | 79.9 ± 32.5 (33.2 – 187) | 0.51 | F |
| Histamine | 0.41 ± 0.04 (0.37 – 0.46) | 0.34 ± 0.14 (0.12 – 0.46) | 0.36 ± 0.12 (0.12 – 0.45) | 0.28 ± 0.21 (0.00 – 0.57) | 0.27 | F |
| Kynurenine | 2.71 ± 0.52 (1.37 – 3.89) | 2.47 ± 0.85 (1.39 – 5.42) | 3.02 ± 0.74 (1.77 – 4.88) | 3.50 ± 2.05 (1.86 – 14.9) | 3e-04 | T |
| Putrescine | 0.09 ± 0.04 (0.03 – 0.20) | 0.11 ± 0.06 (0.02 – 0.32) | 0.09 ± 0.04 (0.03 – 0.21) | 0.14 ± 0.04 (0.06 – 0.23) | 5e-03 | T |
| Serotonin | 0.76 ± 0.35 (0.19 – 1.47) | 0.70 ± 0.40 (0.08 – 1.82) | 0.65 ± 0.38 (0.05 – 1.48) | 0.79 ± 0.47 (0.02 – 1.84) | 0.25 | F |
| Symmetric dimethylarginine | 0.55 ± 0.10 (0.39 – 0.81) | 0.56 ± 0.13 (0.28 – 0.93) | 0.53 ± 0.12 (0.25 – 0.80) | 0.65 ± 0.25 (0.32 – 1.49) | 0.21 | F |
| Taurine | 49.0 ± 18.1 (25.8 – 116) | 90.1 ± 31.6 (32.4 – 172) | 51.4 ± 17.4 (28.2 – 119) | 85.4 ± 31.8 (38.4 – 159) | 2e-15 | T |

**Table S3.** Serum mean levels, standard deviations (SD), and range of biomarker ratios for control subjects (CSs, n=37), first-episode psychosis (FEP) patients at baseline (before treatment with antipsychotics, $FEP_b$, n=52), after 0.6-year treatment ($FEP_{0.6\text{-year}}$, n=44), and after 5.1-year treatment ($FEP_{5.1\text{-year}}$, n=37) with antipsychotics. Comparison between reduced and unrestricted models, *p*-values have been multiple test corrected according to false discovery rate (FDR) method. The unrestricted regression model is designated as True (i.e. more complex set of predictor variables explained more effectively biomarker level alterations over time) or False (i.e. unrestricted model did not provide more explanatory power than a simple one).

| ***Biomarker ratios*** | **CSs** Mean ± SD (range) | **$FEP_b$** Mean ± SD (range) | **$FEP_{(0.6\text{-year})}$** Mean ± SD (range) | **$FEP_{(5.1\text{-year})}$** Mean ± SD (range) | **Comparison between models** | |
|---|---|---|---|---|---|---|
| | | | | | **Adjusted p-value** | **True (T) or false (F)** |
| Alpha-aminoadipic acid / Kynurenine | 0.33 ±0.13 (0.15 – 0.70) | 0.24 ± 0.15 (0.00 – 0.65) | 0.25 ± 0.14 (0.00 – 0.79) | 0.12 ± 0.15 (0.00 – 0.61) | 4e-05 | T |
| Aspartate / Asparagine | 1.07 ± 0.54 (0.43 – 3.07) | 1.11 ± 0.57 (0.36 – 2.71) | 0.98 ± 0.54 (0.27 – 2.44) | 0.38 ± 0.15 (0.15 – 0.77) | 2e-15 | T |
| Glutamate / Glutamine | 1.01 ±1.35 (0.20 – 7.18) | 1.18 ± 1.37 (0.09 – 7.80) | 0.99 ± 0.98 (0.08 – 5.14) | 0.09 ± 0.05 (0.02 – 0.25) | 2e-26 | T |
| Ornithine / Arginine | 0.35 ± 0.11 (0.16 – 0.66) | 0.48 ± 0.20 (0.22 – 1.24) | 0.42 ± 0.13 (0.23 – 0.81) | 0.61 ± 0.21 (0.30 – 1.14) | 2e-06 | T |
| Tyrosine / Phenylalanine | 1.03 ±0.23 (0.49 – 1.57) | 0.86 ± 0.16 (0.61 – 1.28) | 0.95 ± 0.49 (0.37 – 2.36) | 1.01 ± 0.18 (0.61 – 1.32) | 6e-04 | T |

**Table S4.** Estimated effects of complex set of predictor variables on amino acids serum concenrations between control subjects (CSs, n=37), first-episode psychosis (FEP) patients at baseline (before treatment with antipsychotics, $FEP_{b,}$ n=52), after 0.6-year treatment ($FEP_{0.6\text{-year},}$ n=44), and after 5.1-year treatment ($FEP_{5.1\text{-year},}$ n=37) with antipsychotics: results from linear mixed-effects model.

| Amino acids | Intercept | Age | Gender | Body mass index | Smoking status | Disease and treatment effect | | | Time between $FEP_{(b)}$ and $FEP_{(0.6\text{-year})}$ | Time between $FEP_{(0.6\text{-year})}$ and $FEP_{(5.1\text{ year})}$ |
|---|---|---|---|---|---|---|---|---|---|---|
| | | | | | | **FEP patients before treatment** | **FEP patients after 0.6-year treatment** | **FEP patients after 5.1-year treatment** | | |
| | **Estimate (standard error), *p*-value** | | | | | | | | | |
| Alanine | 3.67 (0.24), *p*<1e-04 | -0.001 (0.004), ns | -0.04 (0.05), ns | 0.01 (0.007), ns | 0.09 (0.06), ns | -0.11 (0.06), ns | 0.04 (0.07), ns | 0.10 (0.08), ns | 0.18 (0.62), ns | 0.06 (0.04), ns |
| Arginine | 5.09 (0.16), *p*<1e-04 | 0.005 (0.004), ns | -0.07 (0.05), ns | -0.004 (0.006), ns | -0.02 (0.05), ns | -0.14 (0.06), *p*=0.01 | -0.07 (0.06), ns | -0.18 (0.07), *p*=0.01 | 0.31 (0.56), ns | 0.04 (0.03), ns |
| Asparagine | 4.10 (0.26), *p*<1e-04 | -0.01 (0.006), ns | -0.06 (0.08), ns | -0.008 (0.01), ns | -0.10 (0.09), ns | -0.07 (0.09), ns | -0.003 (0.10), ns | **0.53 (0.11), *p*<1e-04** | 1.88 (0.71), *p*=0.01 | 0.04 (0.05), ns |
| Aspartate | 5.09 (0.16), *p*<1e-04 | -0.005 (0.006), ns | -0.04 (0.07), ns | 0.001 (0.009), ns | -0.14 (0.08), ns | -0.07 (0.09), ns | -0.19 (0.10), ns | **-0.59 (0.11), *p*<1e-04** | -0.68 (0.89), ns | 0.08 (0.05), ns |
| Citrulline | 3.46 (0.21), *p*<1e-04 | -0.0001 (0.005), ns | -0.03 (0.06), ns | -0.007 (0.008), ns | 0.02 (0.06), ns | -0.20 (0.07), *p*=0.006 | -0.06 (0.08), ns | 0.02 (0.09), ns | 1.21 (0.67), ns | 0.05 (0.04), ns |
| Glutamine | 6.37 (0.28), *p*<1e-04 | -0.01 (0.01), *p*=0.03 | 0.06 (0.08), ns | -0.01 (0.01), ns | 0.06 (0.08), ns | 0.02 (0.10), ns | 0.18 (0.11), ns | **1.16 (0.13), *p*<1e-04** | 2.04 (0.80), *p*=0.01 | 0.10 (0.06), ns |
| Glutamate | 4.42 (0.28), *p*<1e-04 | 0.02 (0.01), *p*=0.03 | -0.43 (0.09), *p*<1e-04 | 0.03 (0.01), *p*=0.003 | 0.11 (0.09), ns | 0.002 (0.10), ns | -0.17 (0.10), ns | **-1.50 (0.12), *p*<1e-04** | -1.13 (0.74), ns | -0.07 (0.06), ns |
| Glycine | 5.53 (0.17), *p*<1e-04 | 0.003 (0.004), ns | 0.03 (0.05), ns | -0.006 (0.006), ns | 0.08 (0.05), ns | 0.07 (0.06), ns | 0.11 (0.06), ns | 0.12 (0.07), ns | 0.81 (0.54), ns | -0.005 (0.03), ns |
| Histidine | 4.40 (0.12), *p*<1e-04 | -0.003 (0.003), ns | -0.07 (0.03), *p*=0.04 | 0.01 (0.004), *p*=0.03 | 0.02 (0.04), ns | -0.08 (0.04), ns | 0.02 (0.04), ns | 0.09 (0.05), ns | 0.07 (0.38), ns | -0.002 (0.03), ns |

(continued)

**Table S4.** (continued)

| Amino acids | Intercept | Age | Gender | Body mass index | Smoking status | Disease and treatment effect: FEP patients before treatment | FEP patients after 0.6-year treatment | FEP patients after 5.1-year treatment | Time between $FEP_{(b)}$ and $FEP_{(0.6\text{-}year)}$ | Time between $FEP_{(0.6\text{-}year)}$ and $FEP_{(5.1\ year)}$ |
|---|---|---|---|---|---|---|---|---|---|---|
| | Estimate (standard error), *p*-value | | | | | | | | | |
| Isoleucine | 4.34 (0.17), *p*<1e-04 | -0.005 (0.004), ns | -0.25 (0.05), *p*<1e-04 | 0.02 (0.006), *p*=0.008 | -0.07 (0.05), ns | -0.06 (0.06), ns | -0.03 (0.06), ns | -0.03 (0.07), ns | 0.04 (0.56), ns | 0.03 (0.03), ns |
| Leucine | 5.31 (0.20), *p*<1e-04 | -0.005 (0.004), ns | -0.32 (0.05), *p*<1e-04 | 0.02 (0.007), *p*=0.006 | -0.09 (0.06), ns | -0.06 (0.07), ns | -0.07 (0.07), ns | 0.03 (0.08), ns | -0.005 (0.66), ns | 0.005 (0.04), ns |
| Lysine | 5.13 (0.16), *p*<1e-04 | 0.0002 (0.004), ns | -0.07 (0.05), ns | 0.008 (0.006), ns | 0.003 (0.05), ns | -0.009 (0.06), ns | 0.03 (0.06), ns | 0.07 (0.07), ns | 0.12 (0.52), ns | -0.03 (0.03), ns |
| Methionine | 2.94 (0.31), *p*<1e-04 | -0.03 (0.007), *p*=8e-04 | -0.07 (0.09), ns | -0.002 (0.01), ns | -0.19 (0.10), ns | 0.03 (0.11), ns | 0.25 (0.12), *p*=0.04 | **1.35 (0.14), *p*<1e-04** | 2.37 (0.98), *p*=0.04 | 0.03 (0.07), ns |
| Ornithine | 3.97 (0.21), *p*<1e-04 | 0.00004 (0.005), ns | -0.15 (0.06), *p*=0.04 | 0.004 (0.008), ns | 0.05 (0.06), ns | 0.13 (0.07), ns | 0.09 (0.08), ns | **0.32 (0.09), *p*=9e-04** | 0.65 (0.64), ns | 0.01 (0.04), ns |
| Phenylalanine | 4.21 (0.15), *p*<1e-04 | -0.004 (0.003), ns | -0.10 (0.04), *p*=0.03 | 0.008 (0.005), ns | -0.02 (0.04), ns | 0.02 (0.05), ns | -0.04 (0.06), ns | 0.07 (0.06), ns | -0.21 (0.50), ns | -0.008 (0.03), ns |
| Proline | 5.31 (0.19), *p*<1e-04 | 0.001 (0.005), ns | -0.17 (0.06), *p*=0.004 | 0.005 (0.007), ns | 0.07 (0.06), ns | **-0.25 (0.07), *p*=2e-04** | 0.01 (0.07), ns | 0.01 (0.08), ns | 0.52 (0.61), ns | 0.06 (0.04), ns |
| Serine | 5.19 (0.17), *p*<1e-04 | 0.005 (0.004), ns | -0.01 (0.006), ns | -0.01 (0.006), ns | -0.02 (0.03), ns | 0.03 (0.06), ns | 0.02 (0.06), ns | -0.05 (0.07), ns | 0.29 (0.56), ns | 0.03 (0.03), ns |
| Threonine | 5.15 (0.19), *p*<1e-04 | -0.002 (0.005), ns | 0.005 (0.06), ns | -0.003 (0.007), ns | 0.04 (0.06), ns | -0.12 (0.07), ns | -0.02 (0.07), ns | -0.08 (0.08), ns | 0.22 (0.60), ns | -0.006 (0.04), ns |
| Tryptophan | 4.55 (0.16), *p*<1e-04 | -0.008 (0.004), ns | -0.17 (0.05), *p*=5e-04 | 0.001 (0.006), ns | -0.02 (0.05), ns | -0.12 (0.06), *p*=0.04 | -0.04 (0.06), ns | 0.11 (0.52), ns | 0.09 (0.52), ns | 0.002 (0.03), ns |
| Tyrosine | 4.05 (0.18), *p*<1e-04 | -0.003 (0.004), ns | -0.13 (0.05), *p*=0.01 | 0.01 (0.006), *p*=0.04 | -0.02 (0.05), ns | -0.16 (0.06), *p*=0.01 | -0.05 (0.07), ns | 0.001 (0.08), ns | -0.03 (0.59), ns | 0.04 (0.04), ns |
| Valine | 5.19 (0.16), *p*<1e-04 | -0.002 (0.004), ns | -0.20 (0.05), *p*=1e-04 | 0.02 (0.006), *p*=0.005 | -0.04 (0.05), ns | -0.08 (0.06), ns | -0.02 (0.06), ns | 0.16 (0.07), *p*=0.02 | 0.19 (0.51), ns | 0.02 (0.03), ns |

Significant differences in the biomarker levels over time in the patients group compared to CSs are marked in bold.
ns: $p \geq 0.05$.

**Table S5.** Estimated effects of complex set of predictor variables on biogenic amines serum concenrations between control subjects (CSs, n=37), first-episode psychosis (FEP) patients at baseline (before treatment with antipsychotics, $FEP_{b,}$ n=52), after 0.6-year treatment ($FEP_{0.6\text{-}year,}$ n=44), and after 5.1-year treatment ($FEP_{5.1\text{-}year,}$ n=37) with antipsychotics: results from linear mixed-effects model.

| Biogenic amines | Intercept | Age | Gender | Body mass index | Smoking status | Disease and treatment effect | | | Time between $FEP_{(b)}$ and $FEP_{(0.6\text{-}year)}$ | Time between $FEP_{(0.6\text{-}year)}$ and $FEP_{(5.1\ year)}$ |
|---|---|---|---|---|---|---|---|---|---|---|
| | | | | | | **FEP patients before treatment** | **FEP patients after 0.6-year treatment** | **FEP patients after 5.1-year treatment** | | |
| | **Estimate (standard error), *p*-value** | | | | | | | | | |
| Acetylornithine | -0.05 (0.49), ns | 0.01 (0.01), ns | -0.06 (0.14), ns | 0.02 (0.02), ns | -0.36 (0.15), *p*=0.02 | 0.04 (0.17), ns | -0.01 (0.18), ns | -0.01 (0.21), ns | 0.42 (1.69), ns | -0.09 (0.10), ns |
| Alpha- amino-adipic acid | 0.46 (0.23), ns | 0.002 (0.006), ns | -0.24 (0.07), *p*=4e-04 | 0.02 (0.009), *p*=0.01 | -0.07 (0.07), ns | **-0.37 (0.08), *p*<1e-04** | -0.25 (0.09), *p*=0.006 | **-0.65 (0.10), *p*<1e-04** | 0.81 (0.71), ns | -0.01 (0.05), ns |
| Asymmetric dimethylarginine | -1.00 (0.16), *p*<1e-04 | 0.0004 (0.004), ns | -0.02 (0.05), ns | 0.005 (0.006), ns | 0.08 (0.05), ns | 0.03 (0.06), ns | 0.03 (0.06), ns | 0.23 (0.07), *p*=0.002 | 0.24 (0.50), ns | 0.02 (0.03), ns |
| Creatinine | 4.45 (0.17), *p*<1e-04 | -0.006 (0.004), ns | -0.27 (0.05), *p*<1e-04 | 0.003 (0.006), ns | -0.07 (0.05), ns | -0.02 (0.06), ns | -0.02 (0.06), ns | 0.11 (0.07), ns | 0.23 (0.48), ns | -0.005 (0.04), ns |
| Histamine | 0.53 (0.09), *p*<1e-04 | -0.002 (0.002), ns | -0.03 (0.03), ns | -0.002 (0.003), ns | -0.03 (0.03), ns | -0.06 (0.03), *p*=0.04 | -0.06 (0.03), ns | -0.09 (0.04), *p*=0.03 | 0.19 (0.17), ns | -0.006 (0.02), ns |
| Kynurenine | 0.73 (0.17), *p*=1e-04 | -0.005 (0.004), ns | -0.14 (0.06), *p*=0.006 | 0.02 (0.006), *p*=0.003 | 0.04 (0.05), ns | -0.14 (0.06), *p*=0.02 | 0.04 (0.06), ns | 0.11 (0.08), ns | 0.44 (0.54), ns | -0.07 (0.04), ns |
| Putrescine | -2.59 (0.31), *p*<1e-04 | -0.002 (0.008), ns | -0.17 (0.09), ns | 0.01 (0.01), ns | 0.09 (0.10), ns | -0.003 (0.11), ns | -0.10 (0.11), ns | 0.33 (0.13), *p*=0.02 | -0.26 (0.96), ns | -0.04 (0.06), ns |
| Serotonin | 0.03 (0.45), ns | 0.007 (0.01), ns | -0.13 (0.13), ns | -0.02 (0.02), ns | 0.31 (0.14), *p*=0.03 | -0.24 (0.15), ns | -0.32 (0.17), ns | -0.21 (0.19), ns | 0.72 (1.54), ns | -0.16 (0.09), ns |
| Symmetric dimethylarginine | -0.67 (0.16), *p*=1e-04 | -0.003 (0.004), ns | -0.06 (0.05), ns | 0.007 (0.006), ns | -0.05 (0.05), ns | 0.02 (0.06), ns | -0.04 (0.06), ns | 0.12 (0.07), ns | 0.20 (0.57), ns | -0.01 (0.03), ns |
| Taurine | 3.79 (0.21), *p*<1e-04 | 0.0002 (0.005), ns | -0.03 (0.06), ns | 0.002 (0.008), ns | 0.06 (0.07), ns | **0.59 (0.08), *p*<1e-04** | 0.02 (0.08), ns | **0.51 (0.10), *p*<1e-04** | -0.87 (0.77), ns | -0.03 (0.05), ns |

Significant differences in the biomarker levels over time in the patients group compared to CSs are marked in bold.
ns: $p \geq 0.05$.

**Table S6.** Estimated effects of complex set of predictor variables on biomarker ratios between control subjects (CSs, n=37), first-episode psychosis (FEP) patients at baseline (before treatment with antipsychotics, $FEP_b$, n=52), after 0.6-year treatment ($FEP_{0.6\text{-year}}$, n=44), and after 5.1-year treatment ($FEP_{5.1\text{-year}}$, n=37) with antipsychotics: results from linear mixed-effects model.

| **Ratios of amino acids and biogenic amines** | **Intercept** | **Age** | **Gender** | **Body mass index** | **Smoking status** | **Disease and treatment effect** | | | **Time between $FEP_{(b)}$ and $FEP_{(0.6\text{-year})}$** | **Time between $FEP_{(0.6\text{-year})}$ and $FEP_{(5.1\text{ year})}$** |
|---|---|---|---|---|---|---|---|---|---|---|
| | | | | | | **FEP patients before treatment** | **FEP patients after 0.6-year treatment** | **FEP patients after 5.1-year treatment** | | |
| | **Estimate (standard error), *p*-value** | | | | | | | | | |
| Alpha-amino-adipic acid / Kynurenine | 0.28 (0.09), *p*=0.002 | 0.001 (0.002), ns | -0.05 (0.03), ns | 0.003 (0.003), ns | -0.03 (0.03), ns | -0.10 (0.03), *p*=0.003 | -0.10 (0.04), *p*=0.007 | **-0.23 (0.04), *p*<1e-04** | 0.21 (0.30), ns | 0.02 (0.02), ns |
| Aspartate / Asparagine | -0.37 (0.29), ns | 0.008 (0.007), ns | 0.02 (0.09), ns | 0.006 (0.01), ns | -0.06 (0.09), ns | 0.007 (0.10), ns | -0.20 (0.11), ns | **-1.09 (0.13), *p*<1e-04** | -2.13 (1.02), *p*=0.04 | 0.06 (0.06), ns |
| Glutamate / Glutamine | -1.95 (0.49), *p*=1e-04 | 0.03 (0.01), *p*=0.02 | -0.50 (0.14), *p*=8e-04 | 0.05 (0.02), *p*=0.01 | 0.18 (0.15), ns | -0.01 (0.17), ns | -0.36 (0.18), ns | **-2.65 (0.22), *p*<1e-04** | -3.26 (1.30), *p*=0.01 | -0.14 (0.10), ns |
| Ornithine / Arginine | -1.10 (0.22), *p*<1e-04 | -0.005 (0.005), ns | -0.08 (0.06), ns | 0.007 (0.008), ns | 0.08 (0.07), ns | **0.27 (0.07), *p*=4e-04** | 0.163 (0.08), *p*=0.04 | **0.50 (0.09), *p*<1e-04** | 0.36 (0.66), ns | -0.02 (0.04), ns |
| Tyrosine / Phenylalanine | -0.16 (0.12), ns | 0.001 (0.003), ns | -0.04 (0.03), ns | 0.04 (0.005), ns | 0.007 (0.04), ns | **-0.18 (0.04), *p*=2e-04** | -0.01 (0.05), ns | -0.07 (0.05), ns | -0.008 (0.44), ns | 0.04 (0.03), ns |

Significant differences in the biomarker ratio levels over time in the patients group compared to CSs are marked in bold.

ns: *p*≥0.05.
